# Insulin evokes release of endozepines from astrocytes of the NTS to modulate glucose metabolism in male rats

**DOI:** 10.1016/j.molmet.2025.102255

**Published:** 2025-09-19

**Authors:** Lauryn E. New, Niannian Wang, Holly E. Smith, Ross Birks, Shabbir Khan Afridi, Joanne C. Griffiths, Ryan Hains, Jamie Johnston, Beatrice M. Filippi

**Affiliations:** 1 School of Biomedical sciences, Faculty of Biological Sciences, University of Leeds, Leeds, UK; 2 Current position: Postdoctoral Research Assistant, Queen Mary university, London, UK

**Keywords:** Insulin, Astrocytes, Nucleus of the solitary tract, Glucose metabolism, Endozepines, DBI and ODN

## Abstract

The central nervous system (CNS) plays a key role in regulating metabolic functions, but conditions like obesity and diabetes can disrupt this balance. Within the CNS, the nucleus of the solitary tract (NTS) in the dorsal vagal complex (DVC) senses insulin and regulates feeding behaviour and hepatic glucose production. However, we still know little about which cells in the NTS are sensitive to insulin. We show that in male rats insulin receptors in astrocytes are crucial for the NTS's ability to regulate glucose production in the liver. We demonstrate that insulin evokes the release of endozepines from primary astrocytes and direct infusion of endozepines into the NTS mimics the effects of insulin. Inhibition of the benzodiazepine binding site of GABA_A_ receptors prevents action of both insulin and endozepines. The effect of endozepines within the NTS is mimicked by GABA_A_ antagonists and prevented by an agonist, suggesting that insulin prompts astrocytes to release endozepines, which then attenuate GABA_A_ receptor activity, ultimately reducing glucose production in the liver. We also show that high-fat-diet-induced insulin resistance in the NTS can be circumvented by endozepine administration.

Our study is the first to show that insulin–dependent release of endozepines from NTS-astrocytes is fundamental to control blood glucose levels.

## Highlights

• Insulin receptors in astrocytes are essential for the NTS's regulation of hepatic glucose production.
• Insulin stimulates the release of endozepines (DBI/ODN) from astrocytes, and ODN injection into the NTS reduces hepatic glucose production.
• The action of endozepines in the NTS is mimicked by GABA_A_ antagonists and inhibited by a GABA_A_ agonist.
• Injection of ODN or Gabazine into the NTS of HFD-fed rats bypasses insulin resistance and decreases hepatic glucose production.

## Graphical abstract

**Figure:**
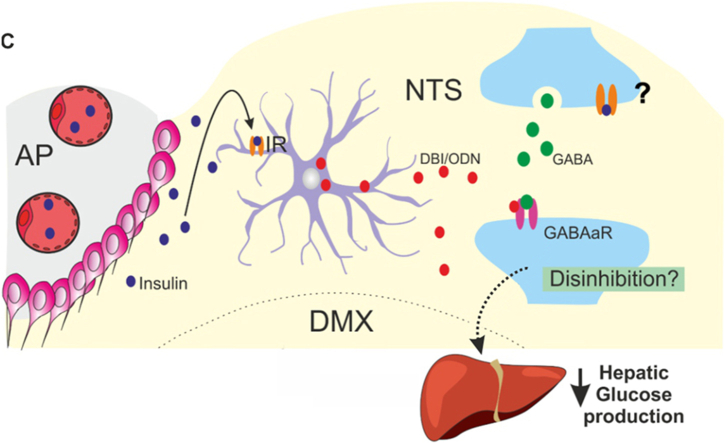

## 1 Introduction

The maintenance of glucose homeostasis requires the fine balance between the release and uptake of glucose by different organs, all coordinated by the modulation of insulin and glucagon levels. The interaction between pancreatic islets and insulin-sensitive tissues has been traditionally used to explain how glucose homeostasis is maintained in the body, but compelling evidence for an important role for the central nervous system (CNS) continues to accumulate [1]. The CNS can sense changes in blood glucose and insulin and other hormones levels and send signals to both sympathetic and parasympathetic nervous system to maintain metabolic homeostasis. Hormones like insulin and leptin act on brain receptors to regulate metabolic balance by modulating the activity of specific neuronal populations [2,3]. Insulin crosses the blood brain barrier and acts on different brain areas to lower plasma glucose levels [4,5] that is in part due to decreased hepatic glucose production (HGP) [6], which is communicated by the vagal nerve efferent pathway [7]. Several brain regions have been identified that sense insulin and regulate HGP, including the arcuate nucleus of the hypothalamus [7] and more recently the Nucleus of the Solitary Tract (NTS) in the Dorsal Vagal Complex (DVC) [8,9]. Insulin modulates neural excitability in these brain regions by triggering the opening the ATP-sensitive potassium channel (K_ATP_) [8,9]; in the arcuate nucleus it results in modulation of both POMC and AGRP neurons, whereas the neural targets of insulin with the NTS have not yet been identified.

The NTS receives sensory information from vagal afferent fibres that carry information from the viscera. The NTS then integrates and relays this information to the hypothalamus, the dorsal motor nucleus of the vagus (DMV) and other brain regions [10]. The hypothalamus regulates food intake and metabolic behaviour, while the DMV signals back to the viscera to coordinate gastric reflexes and to the liver to modulate HGP [11,12]. The NTS contains neurons that release and are sensitive to a large array of neurotransmitters, including γ-aminobutyric acid (GABA) [13], glutamate [14], catecholamines [15], as well as neuropeptides such as glucagon-like peptide 1 (GLP-1) [16] and neuropeptide Y (NPY) [17]. An essential yet unanswered question is identifying the specific NTS cell types involved in regulating food intake and hepatic glucose production (HGP) in response to insulin. Interestingly, astrocytes regulate many aspects of neuronal function, including synaptic plasticity, survival, metabolism, and neurotransmission [18]. Chemogenetic activation or inhibition of NTS astrocytes affects feeding behaviour [19] and inhibiting mitochondrial fission in NTS astrocytes protects rats from high-fat diet-induced insulin resistance and obesity [20]. Intriguingly, specific knockdown of the insulin receptor in astrocytes of the mediobasal hypothalamus (MBH), causes hyperphagia and affects glucose metabolism and energy expenditure [21,22]. Therefore, astrocytes are likely to be important for insulin sensing within the NTS but the specific details have not yet been determined. One mechanism of astrocyte-neuronal cross talk involves the release of astrocyte specific peptides such as endozepines, notably the 86-amino acid precursor diazepam-binding inhibitor (DBI) and its proteolytic cleavage product, octadecaneuropeptide (ODN) which act on GABA_A_ receptors to modulate neuronal activity [[23], [24], [25]]. DBI and its derived peptides have been shown to play a central role in systemic metabolic regulation [26].

Here we investigated the possibility that insulin could act on astrocytes in the NTS to decrease HGP and control blood glucose levels. We demonstrate that insulin can trigger the release of endozepines from astrocytes which modulates GABAergic signalling in the NTS, resulting in decreased hepatic glucose production.

## 2 Results

### 2.1 The insulin receptor is preferentially expressed in astrocytes and GABAergic neurones of the NTS

**Figure 1:**
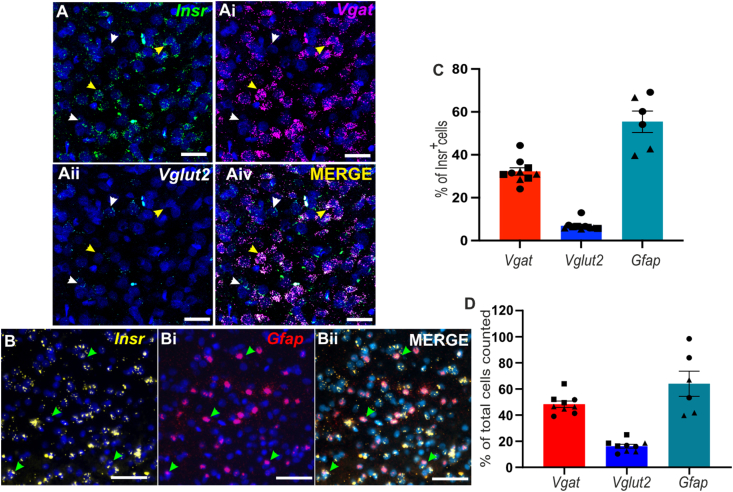
**Cellular population and insulin receptor distribution in the NTS**. **(A and B)** RNAScope staining of the NTS area showing the localization of insulin receptor mRNA (*Insr*, **A** Green and **B**, Yellow) with GABAergic neurones (*Slc32a1*, *Vgat*, **Ai**, Purple), glutamatergic neurones (*Slc12a6*, *Vglut2*, **Aii**, Cyan) and astrocytes (*Gfap,***Bi**, Red). Merged images are shown in **Aiv** and **Bii**. A-AiV Scale bar = 50 μm, B-Bii scale bar = 20 μm. Colocalization with *Insr* is indicated by arrows: yellow (*Insr*^+^ -*Vgat*^*+*^), green *(Insr*^+^ -*Gfap*^+^) and white (*Insr*^+^ -*Vglut*^+^) **(C)** The % of NTS *Insr*^*+*^ cells which were also *Vgat*^+^, *Vglut2*^+^, or *Gfap*^+^ in the NTS. **(D)** The % distribution of astrocytes, GABAergic neurones and glutamatergic neurones. Individual dot shows average 3 ROIs counted from 3 sections per animal, N = 3 animals for *Vgat* and *Vglut2* and 2 for *Gfap (each symbol correspond to 1 animal)*. Image showing the full DVC area and example of ROI taken for analysis is shown in Figure 1A.

The NTS is comprised of a plethora of different neuronal cell types including glutamatergic, GABAergic, and TH-positive neurones among others [27,28]. The lack of information regarding the distribution of the insulin receptor in the NTS prompted us to perform RNAScope focusing on 3 major cell types: astrocytes (*Gfap*), GABAergic (*Slc32a1, Vgat*) and glutamatergic (*Slc12a6, Vglut2*) neurones (Figure 1 A and B). We found that a large fraction of cells within the NTS were positive for the insulin receptor (*Insr*), 64 ± 2.1% of DAPI identified cells. Of these *Insr-*positive cells, 38 ± 2.1 % were GABAergic with only 16 ± 1.5 % being glutamatergic, GABAergic neurons outnumbered glutamatergic by ∼3:1 (Figure 1D). However, the largest population of insulin positive cells were astrocytes (Figure 1 B&C). Overall, these data suggest that when insulin is elevated multiple cell types within the NTS may be modulated at the same time.

### 2.2 Insulin indirectly triggers widespread C-Fos expression in the NTS and directly activates it in astrocytes

**Figure 2:**
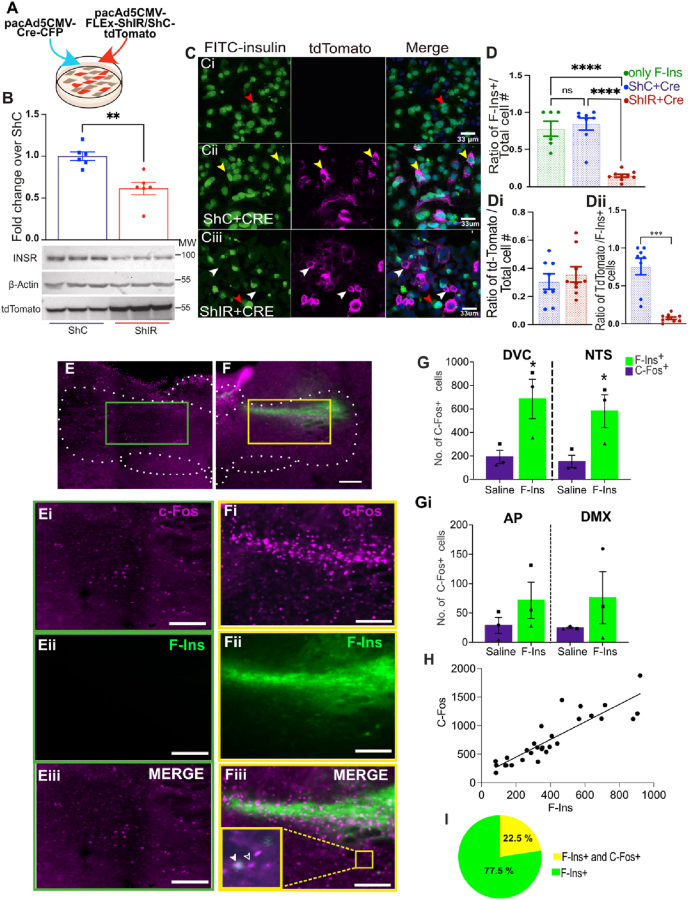
**F-Ins injection increases C-Fos levels in the DVC.** (**A**) PC12 cells co-transfected with a Cre expressing plasmid and a FLEx plasmid expressing the ShRNA for the insulin receptor (ShIR) or a scramble ShRNA (ShC) together with tdTomato. (**B**) Quantification of insulin receptor levels and representative western blot images of insulin receptor, b-actin and tdTomato's levels. (**C-D**) Cells were starved overnight and then treated with 800 nM FITC-insulin (F-Ins). Representative image of F–Ins binding to non-transfected cells (Ci), cells expressing ShC (Cii) or ShIR (Ciii) is shown in **C** and quantification of F–Ins binding is shown in **D-Dii**. Statistical significance was determined with 1 way ANOVA and Tukey's post hoc test in D and T-test in B, Di and Dii. Yellow arrows indicate co-localization between tdTomato and FITC-insulin, white arrows indicate tdTomato-positive cells expressing ShRNA without F-Ins. **(E–I)** Rats were injected either with Saline (E) or F-Ins (F) (protocol schematic in Supplementary Fig. S1B). E) Representative slidescanner images showing immunofluorescence of the DVC areas for C-Fos (Magenta) and F-Ins (Green) staining, and C-Fos and F-Ins merged images in saline-injected rats (green box, E-Eiii) and F-Ins injected rats (yellow box, F-Fiii). Closed arrow denote colocalised cell. Open arrow denote non-colocalised cell. Scale bar = 50 μm. **(G-Gi)** Number of C-Fos positive cells in the DVC or NTS (G) and AP or DMV (Gi) of rats treated either with F-Ins or with saline in the NTS. N = 3 animals (average of at list 4 sections per animal (total of 44 sections for saline and 29 for FITC-Ins). ∗p < 0.05, student's t-test. (**H)** The number of C-FOS positive cells was correlated with the number of F-Ins positive cells (pearson's R^2^ = 0.7775, n = 29, N = 3). **I**) Percentage of total F-Ins + cells which were either F-Ins positive only (green wedge) or F-Ins^+^/C-Fos^+^ (yellow wedge).

We have shown that insulin receptors are expressed by multiple cell types within the NTS (Figure 1). We next sought to address how insulin modulates activity within the NTS. To identify cells with functional insulin receptors we took advantage of FITC-tagged insulin. FITC-insulin acts like normal insulin, triggering AKT activation in PC12 cells (although with lower potency, see Supplementary Fig. S2 A to E). Importantly, when the insulin receptor is activated, it becomes internalised eventually reaching the nucleus [[29], [30], [31], [32]]. We confirmed that FITC-insulin acts in the same way as insulin using PC12 cells transfected with either ShRNA to knockdown the insulin receptor (ShIR) or a scrambled Sh-control (ShC) both plasmids also labelled cells with tdTomato to indicate transfection (Figure 2A). As expected, the ShRNA reduced expression of the insulin receptor in PC12 cells (Figure 2B) and cells transfected with the ShRNA did not uptake FITC-insulin (Purple cells in Figure 2 Cii and D-Dii), whereas FITC-insulin internalisation was evident in both untransfected control cells (Figure 2Ci), untransfected neighbours of transfected ShRNA cells (Red arrows, Figure 2 Ciii and D-Dii) and in cells transfected with the ShC (Figure 2 Cii and D-Dii). In summary, Figure 2A–D demonstrates that FITC-insulin is internalised in an insulin receptor-dependent manner. We then used this tool to investigate the effect of insulin on NTS activity by injecting FITC-insulin into the NTS through a brain cannula (Supplementary Fig. S1b) and then staining for the immediate early gene C-Fos, a marker of neural activity [33]. We found a significant increase in C-Fos when we compared NTS of rats injected with FITC-insulin vs rat injected saline (Figure 2E–G, 581.15 ± 98.7 vs 152.9 ± 37.66 for F-Ins and saline injected animals, respectively. *P* = 0.045). Similar trends were seen for AP and DMV with significantly lower numbers (Figure 2Gi). Furthermore, the number of C-Fos positive cells was correlated with the number of FITC-insulin labelled cells (R^2^ = 0.775, 29 FOVs from N = 3, Figure 2H). These data indicate that insulin markedly increases C-Fos expression within the NTS, which is somewhat surprising as the canonical mode of insulin action is to activate the K_ATP_ channel, thus reducing neural activity [7,8]. Indeed, we found that most FITC-insulin labelled cells were not C-Fos positive, only 22.5% of FITC-insulin labelled cells also expressed C-Fos (Figure 2 I). These data suggest that the increased C-Fos expression observed in Figure 2 F‒G is due to an indirect action of insulin possibly due to disinhibition, via direct inhibition of GABAergic neurons or is a result of some action by the smaller population of cells directly activated by insulin that exerts a net excitatory effect on a larger population of neurons.

**Figure 3:**
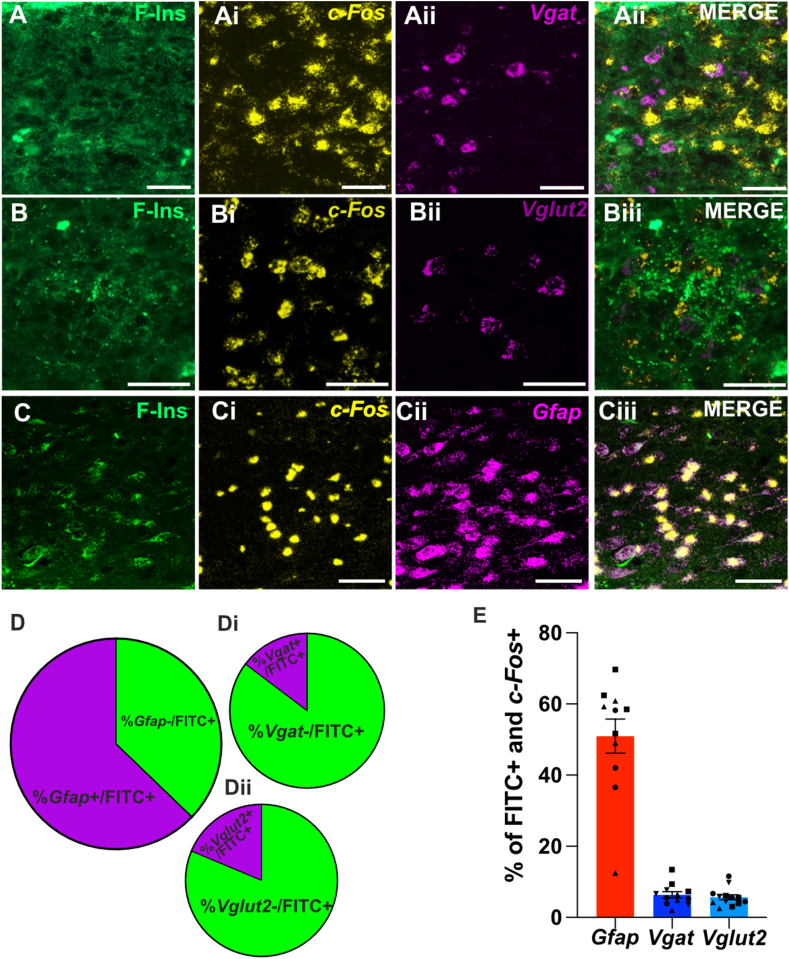
**FITC-Insulin directly activates astrocytes**. **(A-Ciii)** Representative RNAScope staining of the NTS area looking at the localization of FITC-insulin (**A, B and C)** and *c-Fos****(A***i, **Bi** and **Ci**) with *Vgat* (**Aii**), *Vglut2* (**Bii**) or *Gfap* (**Cii**). Merged images are shown in **Aiii and Biii and Ciii**. Scale bar = 50 μm. (**D-Dii)** proportion of F-Ins + cells that are *Gfap (64.36 ± 2.8)* (**D**), *Vgat* (7.99 ± 0.71%) (**Di**) or *Vglut2 (22.94 ± 1.9%)* (**Dii**). (**E)** The % of cell positive for *c-Fos* and FITC-insulin that colocalise with probes for *Gfap, Vgat* or *Vglut2* (average of 3 ROIs counted from 3 sections per animal (*N* = 3 for *Gfap* and N = 4 for *Vgat* and Vglut), each symbol corresponds to 1 animal.

We next sought to determine the cell types in the NTS directly activated by insulin. We combined the FITC-insulin injection, described above, with RNAscope to detect *c-Fos* and the cell type (Figure 3A–C). We first examined the proportion of *vglut2*, *vgat* and *gfap* positive cells that were labelled with FITC-insulin thus indicating the presence of functional insulin receptors. Similar to Figure 1, we found that a large proportion of FITC-insulin labelled cells were astrocytes (*Gfap*+, Figure 3D) and that glutamatergic (*Vglu2*+, Figure 3Di)) neurons contribute only a small fraction. Surprisingly, we found that only a small proportion of FITC-insulin labelled cells were *Vgat* positive (Figure 3Dii). Together with Figure 1 these data show that astrocytes express insulin receptors and appear to have proportionally more functionally active insulin receptors. We also found that astrocytes were the main cell type that was positive for *c-Fos* and FITC-insulin (Figure 3E); 52.8 ± 3% of *c-Fos* and FITC-inulin positive cells were astrocytes, whereas only 6.3 ± 0.8% and 7.8 ± 0.92 % were *Vgat* and *Vglut2* positive, respectively. Together these data show that insulin causes broad changes in C-Fos expression of NTS cell types (Figure 2) and that astrocytes are the cell type that is predominantly directly affected by insulin (Figure 3). Similar induction of C-Fos in astrocytes has been found with other neuropeptides [[34], [35], [36]] and neurotransmitters [37,38].

### 2.3 Knockdown of insulin receptors in NTS-astrocytes impaired insulin-dependent decrease of hepatic glucose production

**Figure 4:**
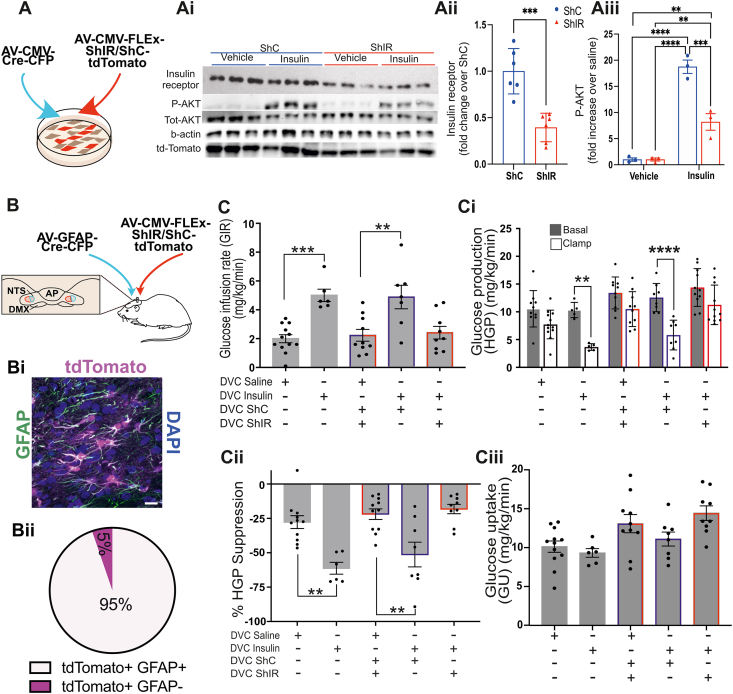
**Effect of the knock down of insulin receptor in NTS GFAP + astrocytes on insulin-dependent regulation of glucose production.** (**A**) Primary astrocytes isolated from rat cortex where infected with 2 adenoviruses, one expressing the Cre-recombinase under the CMV promoter and a FLEx virus expressing the ShIR or the ShC together with tdTomato. Cells where then starved for 5 h and treated with 100 nM insulin or saline for 30 min. Cells where then harvested lysed and analysed by western blotting and probed with the indicated antibodies. The figure shows western blot with insulin receptor and p-AKT quantification normalised for b-actin and total-AKT respectively (Ai-Aiii) statistical analysis was T-test in Aii and 2-way ANOVA with Tukey's post hoc test in Aiii (**B-Ciii**) The day of brain surgery rats were co-injected with an adenovirus expressing the Cre recombinase under the GFAP promoter (GFAP-Cre) and with an adenovirus expressing either the ShIR or the ShC together with tdTomato (B). (Bi) Animals were fixed 4% with PFA and 25 μm coronal sections of the DVC areas were stained with GFAP to visualise astrocytes. tdTomato shows cells expressing the ShRNA for the insulin receptor (ShIR). Nuclei are stained with DAPI. Scale bar = 20 μm (see also Supplementary Fig. S3 C and D for split images and for tdTomato localization in the NTS). (Bii) proportion of tdTomato positive cells colocalizing with GFAP, quantified 9 sections from 3 rats %colocalization = 95.671 ± 1.467 (SEM) (**C-Ciii**) Euglycemic clamp: saline or insulin (a total of 2.52 mU, 1.26 mU r per site) were infused at 0.006 ml/min during the clamp through the DVC catheter (see also Supplementary Fig. S4A). (C) Glucose infusion rate, GIR (mg/Kg/min) during the final 30 min of the clamp for the indicated treatment statistical analysis one way ANOVA. (Ci) Hepatic Glucose production (HGP) (mg/Kg/min) in basal (with square) and clamp (black square) conditions Statistical analysis 2-way ANOVA post hoc Sidak's between basal and clamp in each condition. (Cii) % suppression calculated based on the difference between basal HGP and clamp HGP statistical analysis one way ANOVA. (Giii) Glucose uptake (GU) (mg/Kg/min) during the final 30 min of the clamp for the indicated treatment. Values are shown as mean +SEM; *n* = 12 for saline, *n* = 6 for insulin, *n* = 11 for ShC/ShIR saline (mix of ShIR and ShC rats), *n* = 7 insulin ShC, *n* = 11 insulin ShIR. ∗p < 0.05, ∗∗p < 0.01, ∗∗∗p < 0.001, ∗∗∗∗p < 0.0001.

We have identified that insulin directly activates astrocytes within the NTS, so we next determined whether astrocytic insulin sensing is necessary for the NTS control of the brain-liver axes that modulates blood glucose levels. We developed a series of adenoviruses, two expressing Cre-recombinase tagged with CFP under either the CMV promoter (CMV-Cre) or the GFAP promoter (GFAP-Cre) to specifically target GFAP-expressing astrocytes and two expressing the ShRNA for the insulin receptor (ShIR) or scramble ShRNA (ShC) and tdTomato under 2 lox P sites (Figure 4A,B). Primary astrocytes incubated with CMV-Cre and ShIR showed a 60% reduction of insulin receptor levels and impaired insulin signalling, as shown by decreased AKT phosphorylation (Figure 4A-Aii). We then co-injected the GFAP-Cre viruses and the ShIR or ShC viruses bilaterally (2.5ul each side) into the NTS of rats. The expression of the ShRNA targeting insulin receptors was limited to astrocytes confirmed by post-hoc staining of tdTomato with GFAP (Figure 4Bi-ii and Supplementary Fig. 3). With the insulin receptor selectively knocked down in astrocytes, we then performed a pancreatic euglycemic-basal insulin clamp [8] where rats were injected either with insulin or saline in the NTS and hepatic glucose production (HGP) was measured with the tracer dilution methodology (see Supplementary Fig. S4A). Somatostatin was used to prevent changes in circulating glucoregulatory hormones such as insulin, which was experimentally maintained at basal level (Supplementary Table S1), while tritiated glucose was infused intravenously to measure the amount of glucose that is taken up by peripheral organs or released by the liver (Glucose uptake-GU- and hepatic glucose production-HGP- respectively, Supplementary Fig. S4). The clamp was performed in rats restricted to 70% of their average food intake overnight. We first confirmed that insulin injection into the NTS of wild-type (WT) rats decreases hepatic glucose production (Figure 4C), as previously reported [8]. Next, we investigated whether knocking down the insulin receptor specifically in astrocytes could block this insulin effect. In WT rats or rats expressing scrambled ShRNA in the NTS (ShC), insulin injection—compared to saline-treated controls—significantly increased the glucose infusion rate (GIR) required to maintain basal glycemia (Figure 4C, Supplementary Table S2). This rise in GIR suggests that insulin infusion into the NTS lowers blood glucose levels. To determine whether this drop in blood glucose was due to increased glucose uptake or decreased HGP, we applied tracer dilution methodology [39]. The elevated GIR was associated with reduced HGP (Figure 4Ci), as shown by a significant difference between basal (gray column) and clamp (white column) conditions. This can also be expressed as a % suppression of HGP which was ∼60% in WT rats and ∼50% in ShC rats (Figure 4Cii). Glucose uptake remained unchanged, consistent with previous findings [8] (Figure 4Ciii). Importantly, knocking down the insulin receptor in astrocytes using ShIR abolished this effect, indicating that astrocytes play a major role in the NTS ability to suppress HGP (Figure 4C–Ciii).

### 2.4 Knocking down insulin receptors in NTS astrocytes diminishes the capacity of elevated insulin in the bloodstream to reduce blood glucose levels

**Figure 5:**
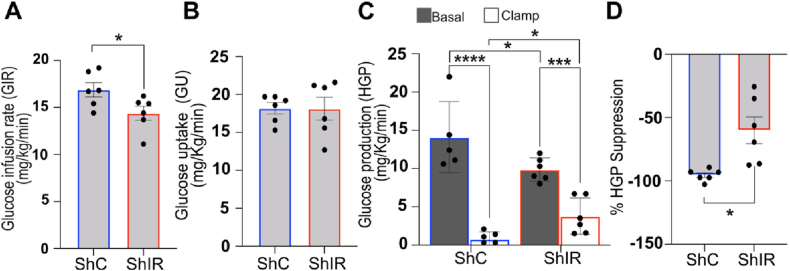
**Effect of knocking down insulin receptors in the NTS on an hyperinsulinemic clamp**. Rats received a continuous injection of 4mU/min/Kg of insulin (see Supplementary Fig. S4B). (**A**) Glucose infusion rate-GIR- (mg/Kg/min) during the final 30 min of the clamp for the indicated treatment. Statistical analysis T-test. (**B**) Glucose uptake -GU- (mg/Kg/min) during the final 30 min of the clamp for the indicated treatment. Statistical analysis T-test. (**C**) Glucose production-HGP-(mg/Kg/min) in basal (with square) and clamp (black square) conditions. Statistical analysis 2-way ANOVA post hoc Sidak's. (**D**) % of HGP suppression. Statistical analysis T-test. Values are shown as mean +SEM; *n* = 6 ShC and 6 ShIR. ∗p < 0.05, ∗∗p < 0.01, ∗∗∗p < 0.001, ∗∗∗∗p < 0.0001.

We have demonstrated that insulin infused into the NTS acts to reduce HGP via astrocytes (Figure 4), we next sought to determine their role in a paradigm closer to normal physiological function. To simulate the post-prandial increase in plasma insulin, we conducted a 2-hour hyperinsulinemic clamp (Supplementary Fig. 4B), increasing insulin levels by 3–4 times. Under hyperinsulinemia, the glucose infusion rate required to maintain euglycemia rose (Figure 5A), indicating a strong inhibition of glucose production (Figure 5C) and a stimulation of glucose uptake (Figure 5B), with nearly 95% suppression of HGP (Figure 5D). However, knocking down insulin receptors in NTS astrocytes significantly reduced the GIR during hyperinsulinemic-euglycemic clamps (Figure 5A). While insulin's ability to stimulate glucose uptake remained unaffected (Figure 5B), suppression of glucose production was partially disrupted (Figure 5C,D). Thus, selective disruption of insulin signalling in NTS astrocytes hampers the suppression of glucose production during elevated plasma insulin. This demonstrates that astrocytic insulin signalling in the NTS will play a role in regulating blood glucose with increased circulating insulin, such as that occurring after meal consumption.

### 2.5 Endozepines are released by astrocytes upon insulin treatment and can decrease HGP

**Figure 6:**
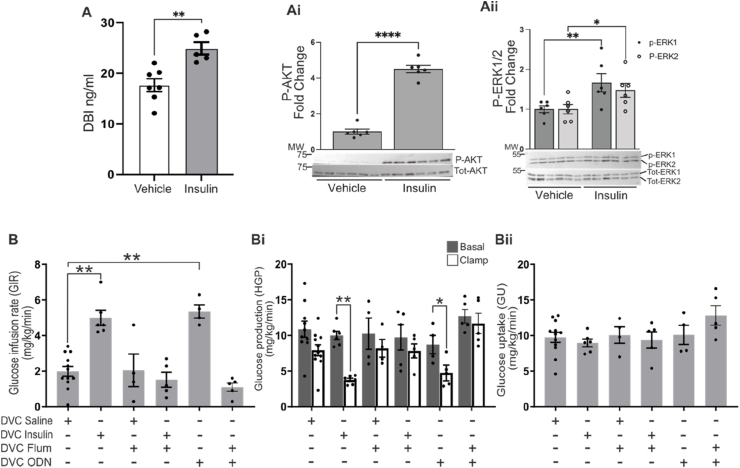
**Endozepines are released by astrocytes upon insulin treatment and can decrease HGP.(A-Aii)** Primary astrocytes were starved for 5 h and treated with 100 nM insulin or saline for 30 min. Cells where then harvested lysed and analysed by western blotting and probed with the indicated antibodies. (A) A comparison of the level of DBI (ng/ml) present in the media from the primary astrocyte experiment treated either with vehicle or insulin as measured by ELISA. Statistical analysis T-Test. (Ai-Aii) Representative western blot with Insulin receptor and p-AKT quantification normalised for b-actin. Statistical analysis 2-way ANOVA post hoc Sidak's between vehicle and insulin. (**B-Bii**) Data from pancreatic euglycemic clamp experiments where either saline, insulin (a total of 2.52 mU, 1.26 mU per site), flumazenil ([33 nM]), ODN ([10 μM]) or flumazenil ([33 nM]) plus ODN ([10 μM]) were infused at 0.006 ml/min during the clamp through the DVC catheter. (**B**) Glucose infusion rate (mg/Kg/min) during the final 30 min of the clamp for the indicated treatment Statistical analysis one way ANOVA post hoc Dunnett's (comparison with DVC Saline). (**Bi**) Glucose production (mg/Kg/min) in basal (with square) and clamp (black square) conditions Statistical analysis 2-way ANOVA post hoc Sidak's between basal and clamp in each condition. (**Bii**) Glucose uptake was comparable in all groups. Values are shown as mean +SEM; *n* = 12 for saline, *n* = 6 insulin, *n* = 4 for flumazenil and 5 for flumazenil plus insulin, *n* = 5 for ODN, *n* = 5 for flumazenil + ODN. ∗p < 0.05, ∗∗p < 0.01, ∗∗∗p < 0.001, ∗∗∗∗p < 0.0001.

We have shown that insulin can directly activate astrocytes within the NTS (Figure 3) and that insulin sensing by astrocytes is required for the NTS to respond to insulin by decreasing HGP (Figure 4, Figure 5). How could NTS astrocytes modulate the activity of NTS neurons to enable communication to the liver? Astrocytes are enriched with the endozepine DBI [25], which, along with its metabolite ODN, can be released from glia and modulate the function of GABA_A_ receptors. Strikingly, insulin treatment of primary astrocyte cultures increased the level of DBI in the media by 40% as measured with an ELISA assay (Figure 6A) and this was accompanied by the expected downstream signalling targets of increased phosphorylation of AKT (Figure 6 Ai) and ERK1/2 (Figure 6 Aii). Importantly, these data demonstrate that insulin triggers the release of endozepines from astrocytes, providing a potential mechanism for astrocytes to influence neural activity within the NTS.

To test whether endozepine signalling in the NTS is required for insulin regulation of HGP, we performed a pancreatic euglycemic-basal insulin clamp using flumazenil, a competitive antagonist of the benzodiazepine binding site of GABA_A_ receptors, that competes with both DBI and ODN acting at this site. Surprisingly, flumazenil treatment prevented the insulin-dependent increase in GIR and decrease HGP (Figure 6B-Bii and S6), indicating endozepine modulation of GABA_A_ receptors in the NTS is required for insulin signalling to the liver. Indeed, direct ODN infusion in the NTS could mimic the effect of insulin, causing an increase of GIR and a decrease of HGP with ∼60% suppression (Figure 6B-Bii and S5). Neither flumazenil nor ODN had any effect on GU (Figure 6Bii). Together our data show that insulin acts directly on astrocytes (Figure 4) which results in release of endozepines (Figure 6A). Moreover, we demonstrate that insulin sensing by NTS astrocytes is required for regulation of HGP (Figure 3C), as is endozepine action on GABA_A_ receptors (Figure 6B). Furthermore, infusion of endozepines into the NTS mimics the action of insulin (Figure 6B).

### 2.6 Inhibition of GABA_A_ receptors in the NTS decreases hepatic glucose production

**Figure 7:**
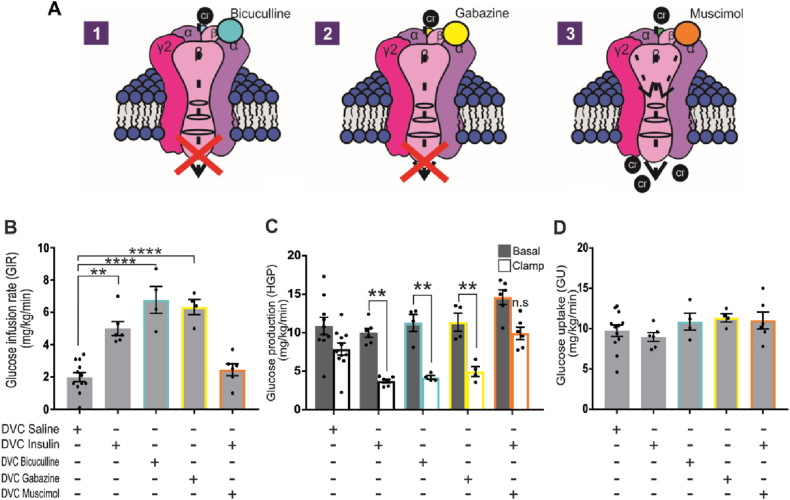
**Effect of GABA inhibition/activation on insulin-dependent regulation of glucose production.** (**A**) schematic representation of bicuculline, gabazine and muscimol mode of action. (**B-D**) Saline, insulin (a total of 2.52 mU, 1.26 mU per site), bicuculline ([1pM)) gabazine ([0.1 mM]) were infused at 0.006 ml/min during the clamp through the DVC catheter. Muscimol ([0.1 μM]) was infused together with insulin 90 min after the experiment started. **(B**) Glucose infusion rate (mg/Kg/min) during the final 30 min of the clamp for the indicated treatment. Statistical analysis 1-way ANOVA post hoc Dunnett's (comparison with DVC Saline) (**C**) Glucose production (mg/Kg/min) in basal (with square) and clamp (black square) conditions. Statistical analysis 2-way ANOVA post hoc Sidak's between basal and clamp in each condition. (**D**) Glucose uptake was comparable in all groups. Values are shown as mean +SEM; *n* = 12 for saline, *n* = 6 insulin, *n* = 4 for Bicuculline and gabazine, *n* = 5 for Muscimol. ∗*p < 0.05; ∗∗p < 0.01; ∗∗∗*p < 0.001; ∗∗∗∗p < 0.0001.

Our data indicate that insulin likely modulates the activity of GABA_A_ receptors via release of endozepines from astrocytes. To determine whether a reduction in GABA_A_ receptor activity in the NTS mimics the effect of insulin in reducing HGP, we used 2 different competitive GABA_A_ antagonists (Figure 7A and Supplementary Fig. S4). Again, insulin injection in the NTS via the brain cannula, increased in GIR associated with a decrease in HGP and a ∼60% suppression of HGP, while glucose uptake was unchanged (Figure 7B–D and S6A). When bicuculine or gabazine was infused into the NTS it mimicked the effect of insulin, there was a clear increase in GIR and this was associated with a clear decrease in HGP and without changes in GU (Figure 7B–D). These data suggest that the endozepines released from astrocytes are acting as negative modulators of NTS GABA_A_ receptors. This is also the first demonstration that attenuating GABA_A_ signalling can reproduce the effect of insulin in the NTS.

### 2.7 GABA_A_ receptor agonism prevents the insulin induced decrease in hepatic glucose production

Our data are consistent with a mechanism whereby insulin in the NTS decreases HGP by acting upon astrocytes, which in turn release endozepines to decrease GABA_A_ receptor activity in neurons. To further test this model we used muscimol, a potent GABA_A_ receptor agonist [40] (Figure 7A) with the idea that directly activating GABA_A_ receptors would prevent any insulin - > astrocyte- > endozepine induced decrease in GABA_A_ activity. In this experiment insulin was infused for 90 min before the addition of muscimol and the start of the clamp (Supplementary Fig. S4). Insulin alone was able to increase GIR and decrease HGP, but in the presence of muscimol, insulin action was completely blocked, we could see a clear decrease in GIR and increase in HGP while GU was not affected (Figure 7z B-D and S6A). Our data indicates that insulin action in the NTS requires negative modulation of GABA_A_ receptors and we can prevent insulin-induced regulation of HGP by activating GABA_A_ receptors in the NTS.

### 2.8 Rescue of NTS control of HGP in insulin-resistant models with gabazine and ODN infusion

**Figure 8:**
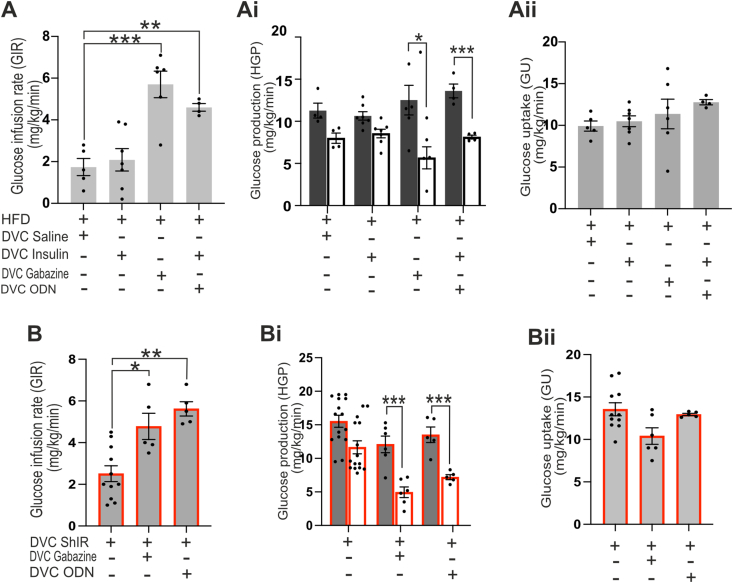
**Gabazine and ODN treatment can recue insulin resistance in astrocytes.** (**A-B**) Saline, insulin (a total of 2.52 mU, 1.26 mU per site), gabazine ([0.1 mM]), or ODN ([10 μM]) were infused at 0.006 ml/min during the clamp through the DVC catheter. Rats were fed with HFD 3 days before the camp to cause insulin resistance in A-Aii. ShIR was used to knock down the insulin receptor in B-Bii. **(A-B**) Glucose infusion rate (mg/Kg/min) during the final 30 min of the clamp for the indicated treatment. Statistical analysis one way ANOVA post hoc Dunnett's (comparison with DVC Saline or ShIR Saline) (**Ai-Bi**) Glucose production (mg/Kg/min) in basal (with square) and clamp (black square) conditions. Statistical analysis 2-way ANOVA post hoc Sidak's between basal and clamp in each condition (**Aii-Bii**) Glucose uptake was comparable in all groups. Values are shown as mean +SEM; *n* = 5 HFD-saline, *n* = 7 HFD-Insulin, *n* = 4 HFD-ODN, *n* = 6 HFD-gabazine, n = 11ShIR-Insulin, n = 5 ShIR + Gabazine and n = 5 ShIR + ODN. ∗*p < 0.05; ∗∗p < 0.01; ∗∗∗*p < 0.001; ∗∗∗∗p < 0.

High fat diet (HFD) feeding causes insulin resistance in the DVC and prevents insulin induced decreases in HGP [8,41]. We hypothesised that using gabazine to inhibit downstream GABAergic signalling could be sufficient to bypass insulin resistance and decrease HGP. To this end we performed a basal glucose clamp study with the same protocol shown in Supplementary Fig. S4 where rats where fed with HFD for 3 days prior to performing the experiment. As expected, in HFD-fed rats, insulin injection in the NTS was not able to increase GIR nor decrease HGP (Figure 8 A‒Aiiand S6B). However, NTS-injection of gabazine was able to increase GIR by lowering HGP in HFD-fed rats (Figure 8 A to Aii and S6B). Similarly, in HFD-fed insulin resistant animals, infusion of ODN was also able increase GIR by lowering HGP (Figure 7B to Bii and S6B). These data suggest that the locus of the HFD-induced insulin resistance is likely astrocytic, as mimicking the downstream effects of insulin on astrocytes rescues the NTS's ability to control HGP. Furthermore, selective knockdown of the insulin receptor in astrocytes recapitulates HFD-induced resistance (Figure 4, Figure 8 B-Bii) and when we either supplied the endozepine ODN or inhibited GABA_A_ receptor with gabazine we again could rescue the effect of insulin (Figure 8 B to Bii and S6Bi).

## 3 Discussion

In summary, we show that astrocytes express insulin receptors (Figure 1, Figure 3) and activation of astrocytic insulin receptors results in the canonical phosphorylation Akt (Figure 4A) as well as inducing *c-Fos* expression (Figure 2E–I) and release of endozepines (Figure 6). We also show that astrocytic insulin sensing is necessary for the NTS's control of hepatic glucose production for both exogenous insulin, directly applied to the NTS (Figure 4), and for increased plasma insulin, as would occur after a meal (Figure 5). Importantly, endozepines applied to the NTS mimic the effect of insulin (Figure 6) and can circumvent HFD induced insulin resistance (Figure 8). Furthermore, we show that insulin and endozepines act via the benzodiazepine site of GABA_A_ receptors (Figure 6) and that insulin and endozepine action in the NTS is mimicked by GABA_A_ inhibition but blocked by GABA_A_ agonism (Figure 7 A). As a result, insulin's net effect will be to reduce GABAergic tone within the NTS, likely resulting in elevated neural activity (Figure 7C), consistent with our finding that insulin increases C-Fos expression, predominantly in F-Ins negative cells (Figure 2, Figure 3).

Our data are consistent with astrocytic endozepines acting as negative allosteric modulators within the NTS and provides a way for insulin to modulate neuronal activity in cells that lack the insulin receptor, for example, if the released endozepines reduce GABA_A_ receptor function this would result in disinhibition of surrounding neurons. Similar negative allosteric actions of endozepines have been found in embryonic spinal cord neurones [42,43], in the sub-ventricular zone [44] and in CA1 pyramidal cells [45]. However, endozepines are also reported to have positive allosteric effects on the GABA_A_ receptor in dorsal root ganglia [46] and the dentate gyrus [45] indicating that endozepine action on GABA_A_ receptors is not yet fully understood. It may depend on the subunit composition of GABA_A_ receptors but clearly depends on the brain area, which is exemplified by Courtney et al. (2018) who showed that within the hippocampus endozepines act as negative modulators within CA1 but positive modulators in the dentate gyrus [45].

Endozepines are known to act at three identified targets. First, the GABA_A_ receptor, which binds both ODN and DBI. Second, a metabotropic G-protein-coupled receptor (GPCR), sensitive to PTX, to which ODN and a smaller fragment, OP, can bind [26]. Third, the Translocator Protein (TSPO), which also binds ODN and DBI [26]. Using flumazenil, a competitive antagonist of the DBI/ODN binding site on GABA_A_ receptors, we showed that this site is critical for insulin signalling in NTS astrocytes; flumazenil injection into the NTS blocked the ODN-dependent reduction in HGP (Figure 6B-Bii). In the hypothalamus, ODN appears to enhance POMC neuronal activity via the metabotropic receptor. OP mimics ODN's effects on feeding and POMC activation, which are blocked by a competitive antagonist of the GPCR, cyclo1-8[D-Leu5]OP [48]. Although ODN also binds GABA_A_ receptors and reduces inhibitory currents in POMC neurons, this alone doesn't activate them [47]. This might suggest that endozepines can regulate glucose and feeding behaviour via separate pathways: GABA_A_ signalling for hepatic control via vagal neurons, and metabotropic signalling for POMC activation and feeding regulation. Indeed, OP activates POMC neurons in the NTS, reducing food intake [48]. Further work is needed to clarify ODN's differential action.

An extensive body of work has shown that endozepines modulate feeding behaviour by acting on both the hypothalamus and brainstem [[49], [50], [51], [52], [53], [54]]. Our findings reveal that insulin stimulation evokes DBI release from astrocytes, and subsequent ODN administration into the NTS effectively reduces HGP. This is the first direct link between insulin signalling and astrocyte-dependent endozepine release. Prior to our study, evidence for brain-derived endozepines in glycemia control was limited. Notably, a 2013 study showed that hypothalamic ODN injection improved glucose tolerance, though the mechanism was unclear [55]. Remarkably, DBI mRNA expression in the hypothalamus [56] is increased by intraperitoneal glucose infusion in fasted rats [40]. Conversely, fasting reduces DBI mRNA in the hypothalamus [54]. These findings suggest that changes in energy status, via elevated insulin, favour increased DBI transcription [56], and our study builds on this by showing insulin evokes DBI release from NTS astrocytes.

Astrocytes are known to release DBI and its derivatives during inflammation and injury, influencing oxidative stress, cell death, and communication with microglia via TSPO-dependent and metabotropic receptor pathways [[57], [58], [59]]. In our findings, however, endozepine release appears to be triggered by insulin independently of classical inflammatory signals. While we cannot entirely exclude subtle inflammatory contributions, the evidence suggests that this pathway primarily involves GABAa receptors and may reflect a metabolic rather than an inflammatory response. Moreover, other metabolic stimuli—such as PACAP [25], cortisol [60], and fasting [61] —also promote endozepine release, supporting a broader role for these peptides in astrocyte-neuron signalling beyond inflammation. Further studies are needed to elucidate how insulin triggers the release of endozepines. Previous research has implicated calcium in the stimulus-evoked release of endozepines [62,63], although transporter-based mechanisms have also been proposed [57]. However, the precise mechanism remains unclear.

Although our study has focused on the effect of insulin receptors in astrocytes, it is worth noting that the insulin receptor is also present in neurons, as illustrated in Figure 1. Whether a direct action of insulin on neurons plays a significant role in regulating HGP remains unclear. There is a clear body of evidence, including our previous work that suggest that insulin activates the K_ATP_ channel to decrease HPG [7,8]. Intriguingly we have shown that the majority of the neurons that uptake insulin are not activated (Fig. 2I), consistent with the hyperpolarising effect of activating K_ATP_ channels. Perhaps, across the population of NTS neurons, insulin acts as a toggle switch, dialling down activity in neurons containing the insulin receptor and K_ATP_ channels, while boosting activity in other neurons by dialling down the effects of GABAergic inhibition with astrocytic release of DBI/ODN. Further investigation should aim to elucidate the effects of insulin and DBI/ODN on NTS neuronal subtypes.

Studies in mouse brain slices show that insulin directly affects glutamatergic terminals in the DMV, a brain area receiving inputs from the NTS that contains the cell bodies of vagal efferent fibres. Insulin reduced the frequency of spontaneous EPSCs in the DMV by inhibiting excitatory stimulation via PI3K-dependent K_ATP_ channel activation. The effects were mediated by receptors on glutamatergic afferent terminals synapsing on DMV neurons [64]. While the source of glutamatergic inputs remains unclear, the hyperpolarizing effect of insulin is a key factor in its influence on neurons. Interestingly, most NTS inputs to the DMV are inhibitory, originating from GABAergic neurons. In the presence of forskolin, which elevates cAMP in GABAergic terminals, insulin decreased inhibitory neurotransmission in normoglycemic, but not hyperglycaemic STZ rats [65]. This suggests a potential communication mechanism where insulin signalling in NTS (maybe via endozepines release) inhibits GABAergic neurons thus decreasing inhibitory control of the DMV.

Short term HFD-feeding in rodents causes insulin resistance and impairs the ability of the DVC and mediobasal hypothalamus to respond to insulin, preventing regulation of HGP and food intake [8,9,66]. Interestingly, in both brain areas, HFD causes alteration of mitochondrial dynamics and increases in ER-stress, these events in turn lead to loss of insulin sensitivity and alter the ability of insulin to regulate blood glucose levels and feeding behaviour [20,41,67,68]. This is consistent with our previous work where we showed that, the development of insulin resistance in the NTS is prevented by inhibiting mitochondrial fragmentation in NTS-astrocytes [20,69], reinforcing the idea that astrocytes are key insulin sensors in the NTS. Indeed, here we have shown that in HFD-fed insulin-resistant rats or in rats where the insulin receptor has been knocked down in the NTS, mimicking the downstream effects of astrocytes by infusion of ODN or gabazine (Figure 8) can bypass the insulin resistance and decrease HGP.

The hyperinsulinemic clamp experiments reinforce the importance of insulin sensing in NTS astrocyte in systemic glucose balance. Knocking down insulin receptors in NTS astrocytes leads to partial loss of HGP suppression, one of the key mechanisms for preventing hyperglycaemia. This also indicates that the NTS can sese changes in systemic insulin levels. The question that remains unanswered is whether our finding in rodent can be related to humans. Studies in humans, mainly using intranasal insulin, show that it influences feeding behaviour, glucose regulation, and cognitive enhancement through central nervous system mechanisms [[70], [71], [72]]. While the mechanism of action is not fully understood, Heni et al. (2014) observed a decrease in glucose infusion rate and increased blood flow in the hypothalamus after intranasal insulin administration [73]. Although it's unclear if the NTS is targeted by intranasal insulin, they also found an increase in high-frequency heart rate (Heart rate variability), which are linked to enhanced parasympathetic activity, likely driven by the NTS via baroreflex activation [74,75].

The physiological relevance of brain insulin action is often questioned due to clamp studies using somatostatin to suppress pancreatic insulin secretion, which results in a lower insulin concentration in the portal vein [76]. This may enhance the impact of extrahepatic insulin action, including that of the brain, which might be less prominent under normal conditions when insulin is delivered directly to the portal vein [76,77]. However, in pathological states such as diabetes or pancreatic failure, portal insulin delivery is impaired. In these cases, alternative pathways—like brain-mediated insulin action—may become essential for glucose regulation.

All experiments were conducted in male rats due to known sex-specific differences in insulin responsiveness [71,72,78]. Future studies should aim to determine whether insulin can also act on the nucleus tractus solitarius (NTS) to reduce hepatic glucose production (HGP) in females, and whether endozepines play a similarly important role in this process.

In summary, our research is beginning to unravel the intricate molecular and cellular events underlying insulin's action in the NTS-mediated modulation of glucose levels. Astrocytes have been identified as the primary site of insulin action, and the release of endozepines appears to play a crucial role in modulating GABAergic signalling to regulate glucose production. Further investigation is warranted to elucidate the precise mechanisms and contribution of insulin-dependent astrocyte-neuronal crosstalk among different neuronal populations within the NTS.

## 4 Materials and methods

### 4.1 Animal preparation

Our studies utilized nine-week-old male Sprague–Dawley rats, weighing between 280 and 320 g, obtained from Charles River Laboratories, (Margate, UK). Animals were used in line with the United Kingdom animals (Scientific Procedures) Act 1986 and ethical standards set by the University of Leeds Ethical Review Committee. Animals were housed individually in cages and maintained under a standard 12-hour light–dark cycle with ad libitum access to standard rat chow or high fat diet and water. On day 0 rats underwent stereotactic surgery (World Precision Instruments)) for implantation of bilateral catheters (Plastics One, Virginia, USA) targeting the nucleus of the solitary tract (NTS) within the dorsal vagal complex (DVC) (coordinates: 0.0 mm on occipital crest, 0.4 mm lateral to midline, and 7.9 mm below the skull surface) [8].

For rats in the GFAP-ShIR cohort, on the day of brain surgery, rats were co-injected with an adenovirus expressing the Cre recombinase under the GFAP promoter (GFAP-Cre) and with an adenovirus expressing a FLEx plasmid where ShRNA for the insulin receptor and tdTomato (ShIR) are cloned within 2 LoxP sites. A scrambled version of the ShRNA was used as a control (ShC).

For animals undergoing pancreatic-euglycemic clamp or hyperinsulinemic clamp, following a one-week recovery period, the rats underwent intravenous catheterization, with catheters inserted into the internal jugular vein and carotid artery for infusion and sampling purposes (Fig. S4).

For this study we used a total of 131 male rats N of rats per group is specified in the figure legends.

### 4.2 Virus preparation

To knock down the insulin receptor in astrocytes we utilized a Cre-LoxP system where pAAV[FLEXon]-CMV > LL:rev(tdTomato:rInsr[miR30-hRNA#3]):rev(LL):WPRE and pAAV[FLEXon]-CMV > LL:rev(tdTomato:Scramble[miR30-shRNA#1]):rev(LL):WPRE control scramble vectors were designed and purchased from Vector Builder. ShRNA (short hairpin RNA) was designed to silence target mRNA of insulin receptor (ShIR), and cloned into an adenoviral shuttle vector to make pacAd5CMV-ShIR-tdTomato and pacAd5CMV-ShC-tdTomato, the scrambled RNA possesses the same nucleotide composition as the ShIR but is incongruent with any target mRNA of IR. This was used as control vector and named as ShC. In addition, a pacAd5CMV-CRECFP vector was designed and generated to introduce Cre recombinase. To produce an adenoviral system expressing the Cre under the GFAP promoter, the CMV promoter was removed from the pacAD5 shuttle vector, and replaced with the rat GFAP promoter. Recombinant adenoviruses were amplified in HEK 293 AD cells and purification was performed using a sucrose-based method [20]. Adenoviruses injected into the animals' brains had a titre between 3 × 10^9^ pfu/ml and 4.4 × 10^11^ pfu/ml.

### 4.3 *In vivo* FITC-insulin DVC infusion

Animals were fitted with a bilateral DVC cannula as detailed above and were allowed to recover for 1 week. Following recovery, animals were injected with 495uM FITC-insulin (2uL per site) into the NTS, the cannula was left in place for 5 min before being removed and replaced with a dummy cannula. After 46 min animals were terminally anaesthetized and perfused with 4% PFA, brains were dissected and prepared for either RNAscope or immunofluorescence (Fig. S1) as detailed below.

### 4.4 RNAScope and immunofluorescence

Animals were terminally anaesthetized (Pentoject, 60 mg/kg IP) and transcardially perfused with 4% paraformaldehyde. The brainstem area containing the DVC was taken and frozen in optimal cutting temperature compound (OCT, VWR international); 10 μm and 25 μm sections were cut using a cryostat for RNAScope and immunofluorescence, respectively.

FITC-insulin + sections were permeabilised in 0.3% PBST (1X PBS, 0.3% Triton X-100 (PBST)) for 10 min and blocked for 1 h with 10% normal donkey serum (Merck Millipore), 1% bovine serum albumin (BSA) (Sigma Aldrich) in 0.3 M glycine (Sigma Aldrich) PBS to prevent nonspecific background labelling. Sections were then incubated C-Fos (1:400 in 0.1% PBST, Cell Signaling Technology, #2250) overnight at 4 °C. After 24 h sections were washed in PBS and then incubated with Alexa Fluor® 555 donkey anti-rabbit (1:1000 in PBS, Thermofisher, #A32794) for 2 h at room temperature, Following PBS wash, sections were mounted using Vectashield plus DAPI (Vector laboratories, H-1200-10). Images were taken with Zen AxioScan V1 slidescanner (Figure 2E,F) and analyzed using cell counter plug in in Fiji (Image J [79]). For each image all the C-Fos positive cells in the DVC area of 1 hemisphere where counted.

RNAScope Multiplex Assay V2 (Bio-Techne) was performed according to manufacturer's instructions in order to characterise the expression of *Insr* (probe cat# 406421) or FITC-insulin with or without *c-Fos* (cat# 403591) in either *Gfap*+ (cat# 407,881-C2) astrocytes, *Vgat*+ (cat# 424541-C3) inhibitory neurones, or *Vglut*+ (cat# 317011-C2) excitatory neurones. Sections were stained with DAPI to label nuclei and imaged using either Zen AxioScan V1 slidescanner (Figure 1) or LSM880 upright confocal microscope (Figure 1, Figure 3). Images were analysed using the cell counter plug in in Fiji (Image J [79]). Cells positive for *Insr* plus *c-Fos*, *Vgat*, *Vglut*, or *Gfap* were counted in 3 regions of interest (200X200 ROI) randomly placed within the NTS from 3 sections containing the DVC from 3 animals (see Supplementary Fig. 1), giving a total of 9 regions of interest per section per animal to give a total of 27 ROIs analysed. Section where selected where AP, NTS and DMX where clearly visible to have the largest NTS area.

To determine the specificity of the GFAP-ShIR-tdTomato expression sections were checked for the presence of tdTomato in the DVC and co-labelled with GFAP (1:1000 in 0.1% PBST, Abcam ab7260) at 4 °C overnight. Following incubation with appropriate secondary antibody (Thermofisher, #A32794) sections were mounted using vectashield plus DAPI. Images were taken using a Zeiss LSM880 upright confocal laser scanning microscope.

### 4.5 DVC infusion and pancreatic-euglycemic/Hyperisnulenimic clamp studies

Four days following intravenous catheterization, animals whose food intake and body weight had returned to within 10% of baseline levels underwent the clamp studies (Fig. S4). To ensure consistent nutritional status during the clamps, rats were limited 70 % of their normal food intake on the night before the experiment. The clamps lasted for 210 min. During the clamp period (from time 0 to 210), saline, insulin (totaling 1.26 μU per site) [8], bicuculline ([1pM]) [80], gabazine ([0.1 mM]) [81], or ODN ([10 μM ∼ 28 ng per side]) [55] were infused at 0.006 ml/min during the clamp through the DVC catheter. Muscimol ([0.1 μM])) [82] was infused together with insulin 90 min after the experiment started. For experiments using the CBR site antagonist flumanzeil, flumazenil ([33 nM]) was pre-infused at 0.006 μl/min for 1 h and then co-infused with insulin or ODN during the clamp. Additionally, a primed-continuous intravenous infusion of [3-3H]glucose (40 μCi bolus, 0.4 μCi/min) was initiated at 0 min and maintained throughout the study to assess glucose kinetics. A pancreatic (basal insulin)-euglycemic clamp was initiated at 90 min and continued until 210 min. Intravenous infusion of somatostatin (3 μg/kg/min) along with insulin (0.7–1 mU/kg/min) was performed to return insulin levels to basal levels. For HFD fed animals insulin was infused at 1.4–1.8mU/kg/min, due to their slightly higher basal insulin levels. For the hyperinsulinemic clamp, insulin was intra-venously infused at 4 mU/Kg/min [8]. A 25% glucose solution was administered starting at 90 min and periodically adjusted to maintain plasma glucose levels. Samples for the determination of [3-3H]glucose-specific activity and insulin levels were collected at 10-minute intervals.

### 4.6 Biochemical analysis

Plasma glucose concentrations were measured by the glucose oxidase method (Glucose Analyzer GM9; Analox Instruments, Stourbridge, UK). Plasma insulin levels were determined by an ultra-sensitive rat insulin ELISA low range assay (0.1–6.4 ng/ml) (Crystal Chem, Elk Grove, IL, USA) used according to manufacturer's instructions.

### 4.7 PC12 cell culture, insulin receptor knockdown, and FITC-insulin treatment

Pheochromocytoma (PC12) cells (AddexBio #C0032002) were cultured in F–12K medium (Gibco # 21127–022) with 15% horse serum (Gibco # 1011–07), 2.5% fetal bovine serum (Gibco # 2024–01), and 1% Penicillin Streptavidin (Sigma #P0781) under 37 °C, 5% CO2 conditions. A confluent 75 mm flask of PC12 cells was diluted 1:3 and plated in a 10 cm dish containing a multi-test 8-well slide (MP Biomedicals # 20190128). 24 h later cells were transfected with 5 μg pacAd5CMV-CRECFP and 5 μg pacAd5CMV-ShIR-tdTomato or 5 μg pacAd5CMV-ShC-tdTomato control (both flexed constructs that express tdTomato and the ShIR/ShC only in the presence of Cre-recombinase) by using GenJet reagent (SL00489-PC12). Fresh complete media was added to the cells after 24 h 48 h later, cells were incubated with 15 uL of 800 nM FITC-insulin for 15 min before being fixed with 4% PFA and then washed with 1 X PBS 3 times. Excess liquid was then aspirated, and the slide left to dry. Cover slips were added and sealed with Vectashield mounting medium plus 4’,6-diamindino-2-phenylindole (DAPI) to visualise nuclei (Vector Laboratories, Burlingame CA, USA). Slides were imaged with an upright confocal microscope (Zeiss LSM880).

### 4.8 Primary astrocyte culture, insulin receptor knockdown, and insulin treatment

Primary astrocytes where isolated following a well-established protocol [83,84]. Rat pups (postnatal day 1–3) were decapitated and the cortices were dissected and centrifuged at 1000rpm for 5 min. The supernatant was removed, 2 ml of trypsin was added and left to incubate for 30 min 2 mg/ml DNase was added and samples were centrifuged for 5 min at 1000g. Once the supernatant was removed 10 ml of culture media (10% FBS, DMEM, 1% PSF) was added and the cells were suspended by repeated pipetting and placed in a Poly-d-lysine coated T75 flask. The following day media was changed and 10 uL PLX was added. One week later, when cells were confluent, flasks were placed on an orbital shaker at 180rpm for 30 min to detach microglia. The microglia containing media was removed and replaced with fresh media and flasks were left on an orbital shaker at 240 rpm for 6 h to remove oligodendrocyte precursor cells. The culture media was removed and flasks were rinsed with PBS twice and trypsin added to detach the astrocytes. Astrocytes were pelleted and then resuspended in 40 ml of fresh astrocyte medium and plated into a Poly-d-lysine coated T75 flask.

For insulin receptor knock down 3–4 days prior to insulin treatment primary astrocytes where infected with 2 adenoviruses expressing Cre-recombinase and a FLEx virus expressing the ShIR or the ShC. Cells where then starved for 5 h and treated with 100 nM insulin or saline for 30 min. Cells where then harvested, lysed and analysed by western blotting, and probed with the indicated antibodies as detailed below.

### 4.9 Western blotting

PC12 or primary astrocyte cells were lysed in lysis buffer (50 mM Tris–Hcl pH 7.5, 1 mM EGTA, 1 mM EDTA, 1% (w/v) NP-40, 1 mM sodium orthovanadate, 50 mM sodium fluoride, 5 mM sodium pyrophosphate, 0.27M sucrose, 1 μM DTT and Pierce Protease Inhibitor Tablets), using a homogeniser on ice. Samples were centrifuged at 12,000 RPM for 15 min at 4° supernatants were collected and protein concentration was determined by using a Bradford Assay. Proteins were separated and measured by western blotting as previously described [20]. Membranes were blocked in 5% BSA TBST and incubated in the primary antbodies anti-INSR (Cell Signaling Technology, #3025), anti-TdTomato (SICGEN, AB8181-200), anti-p-AKT (Cell Signaling Technology, #9271) anti-T-AKT (Cell Signaling Technology, #9272S), anti-β-actin (Cell Signaling Technology, #3700) overnight at 4 °C, followed by incubation with species appropriate secondary HRP-conjugated antibodies (Invitrogen, Life Technologies) in 5% skimmed milk. Membranes were imaged using ECL (BioRad Clarity) with the iBright FL1500 Imaging system and analysed using iBright Analysis software (Thermofisher).

### 4.10 Statistical analysis

All data are presented as mean ± SEM and where applicable data from each animal is reported as a single data point. Analysis was performed using GraphPad Prism 9 software. Statistical significance was assessed using multiple T-tests or one/two-way ANOVA (with Sidak's post-hoc test). “*N*” denotes the number of animals used. Each animal received only 1 type of treatment and comparison was done in between subjects. A p-value less than 0.05 was considered statistically significant. Levels of significance were categorized as follows: (∗*) p < 0.05; (∗∗) p < 0.01; (∗∗∗*) p < 0.001; (∗∗∗∗) p < 0.0001.

## CRediT authorship contribution statement

**Lauryn E. New:** Writing – original draft, Validation, Methodology, Investigation, Formal analysis, Data curation. **Niannian Wang:** Methodology, Formal analysis, Data curation. **Holly E. Smith:** Methodology, Investigation, Formal analysis. **Ross Birks:** Methodology, Formal analysis. **Shabbir Khan Afridi:** Methodology, Formal analysis. **Joanne C. Griffiths:** Validation, Methodology. **Ryan Hains:** Methodology, Formal analysis. **Jamie Johnston:** Writing – review & editing, Supervision, Data curation. **Beatrice M. Filippi:** Writing – review & editing, Writing – original draft, Supervision, Project administration, Funding acquisition, Formal analysis, Data curation, Conceptualization.

## Declaration of Generative AI and AI-assisted technologies in the writing process

During the preparation of this work the authors used Copilot in order to improve language and readability of the manuscript. After using this tool/service, the authors reviewed and edited the content as needed and take full responsibility for the content of the publication.

## Declaration of competing interest

No conflict of interest.

## Data Availability

Data will be made available on request.
