# Supplementary material for: Insulin evokes release of endozepines from astrocytes of the NTS to modulate glucose metabolism in male rats
Source: Mol Metab. 2025 Sep 19;101:102255. doi: 10.1016/j.molmet.2025.102255 (PMC12510046; doi:10.1016/j.molmet.2025.102255)
Supplement: Multimedia component 1 [file mmc1.pdf]

# Insulin evokes release of endozepines from astrocytes of the NTS to modulate glucose metabolism in male rats

Lauryn E. New[1], Niannian Wang[1,2], Holly E. Smith[1], Ross Birks[1], Shabbir Khan Afridi[1], Joanne C. Griffiths[1], Ryan Hains[1], Jamie Johnston[1] and Beatrice M. Filippi[1,3]

1. School of Biomedical sciences, Faculty of Biological Sciences, University of Leeds, Leeds (UK)
2. Current position: Postdoctoral Research Assistant, Queen Mary university, London (UK)
3. Correspondence to: b.m.filippi@leeds.ac.uk

# Supplementary material

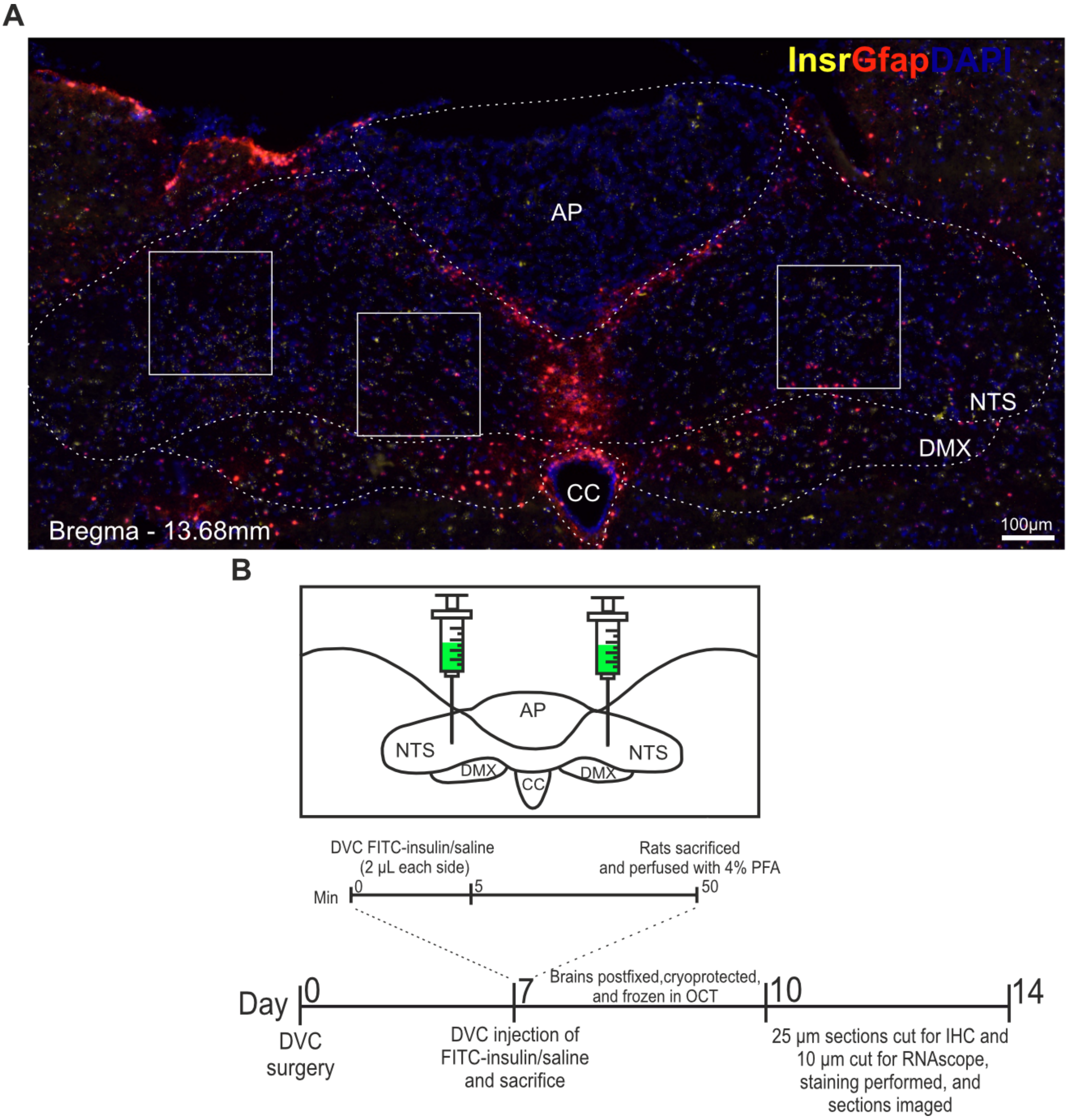


**Figure S1: A) Representative RNASocpe image** showing the full DVC area and an example of how the ROI in figure 1 where selected. **B) Schematic of FITC-insulin NTS injection** paradigm related to figure 2.

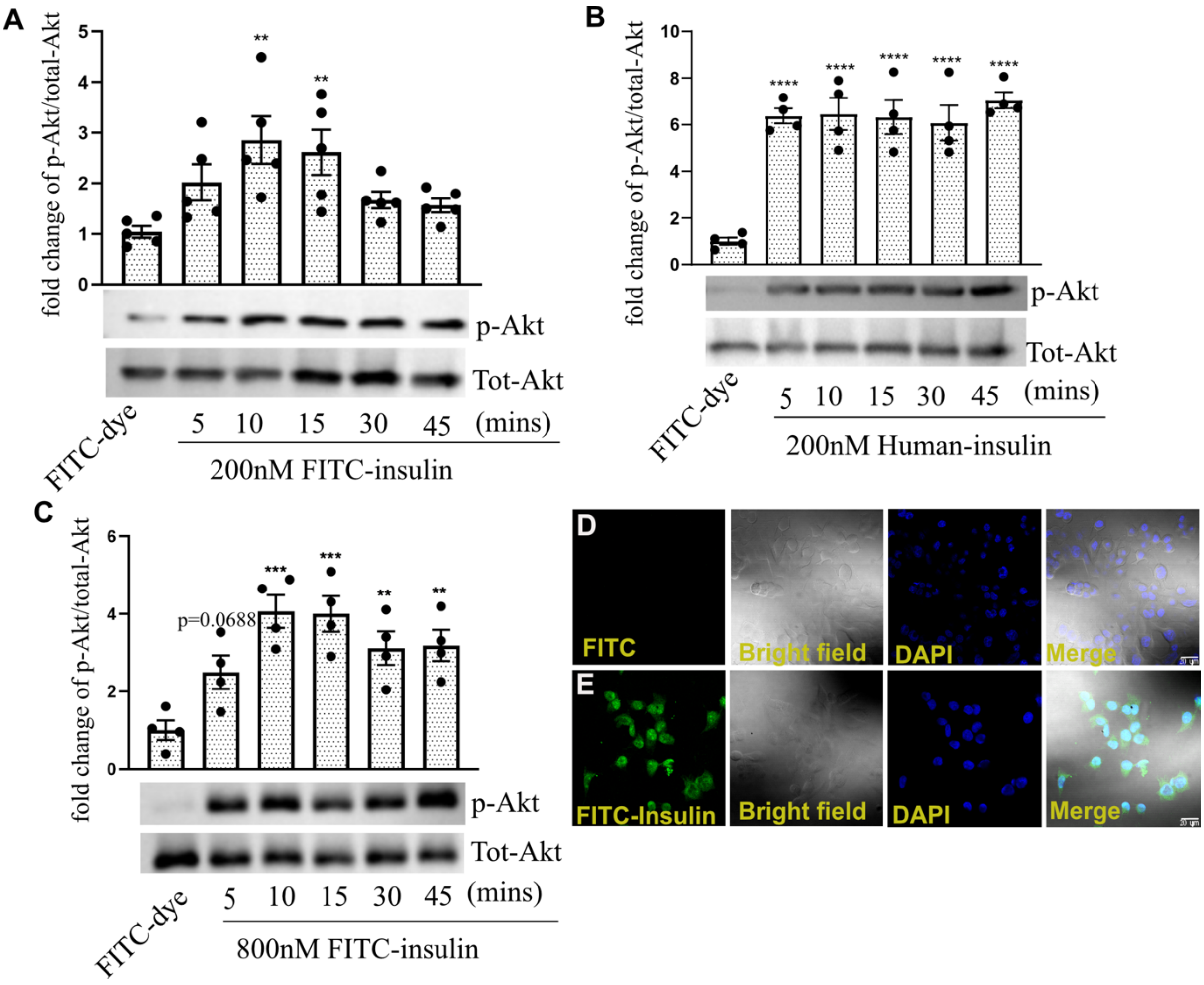


**Figure S2: Comparison of the effect of human insulin and FITC-insulin treatment on AKT activation in PC12 cells.** (**A**) Phosphorylation levels of Akt on Ser473 after insulin (200 nM) treatment at different time points. (**B-C**) Phosphorylation levels of Akt on Ser473 after low FITC-insulin (200 nM) concentration and high FITC-insulin (800 nM) concentration treatment at different time points. FITC-insulin may have multiple FITC molecules attached to a single insulin molecule, making it difficult to calculate the exact concentration. Part of the discrepancy between the two forms of insulin could be because at 200 nM of FITC-insulin, the actual concentration of insulin is likely lower. Indeed when we repeated the same experiment with higher concentration of FITC-insulin (800nM), we could see 2.5 fold increase in p-AKT after 5 min and 4-fold increase after 10- and 15-min. p-AKT was still 3-fold elevated after 30-45 min. 1 way ANOVA with Dunnett post hock test was used to determine significance between (FITC- dye and different times treatment). * $p<0.05$, ** $p<0.01$, *** $p<0.001$.

(**D-E**) Only FITC- insulin is uptake by PC12 cells. PC12 cells were either treated with FITC-dye only or with FITC-insulin (800 nM) for 15 min, only FITC-insulin was taken up by the cells.

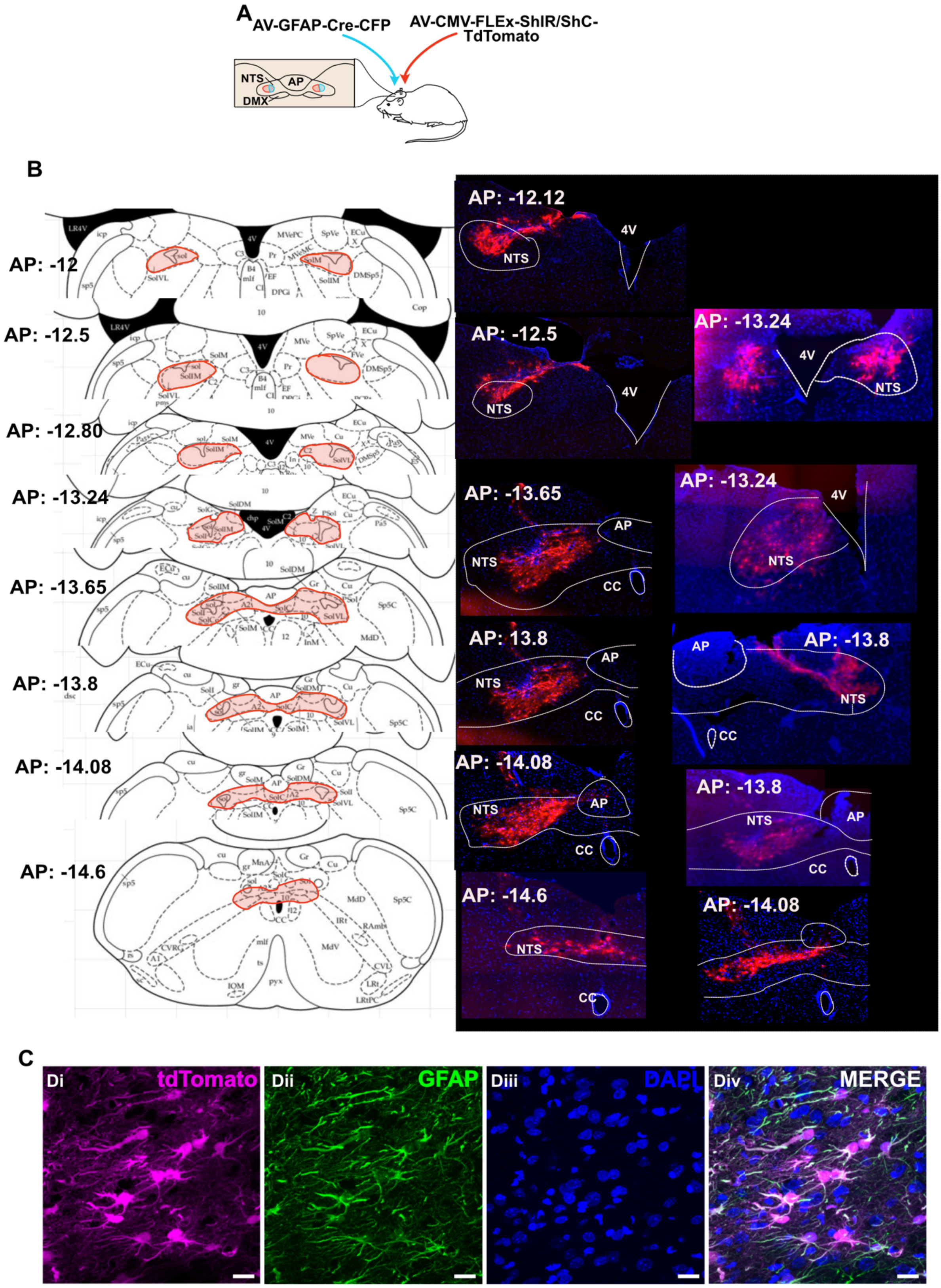


**Figure S3: Expression of ShIR and ShC *in vivo*. A)** Schematic representation of in vivo viral injection**. B)** Representative images showing localization of tdTomato expression after viral injection in the NTS. Atlas images with NTS (Sol) highlighted in red are shown

on the left while representative images of tdTomato localization with corresponding AP are shown in the right**. C)** Representative confocal image of expression of tdTomato+ ShIR in GFAP+ astrocytes in the NTS – related to figure 4Bi.

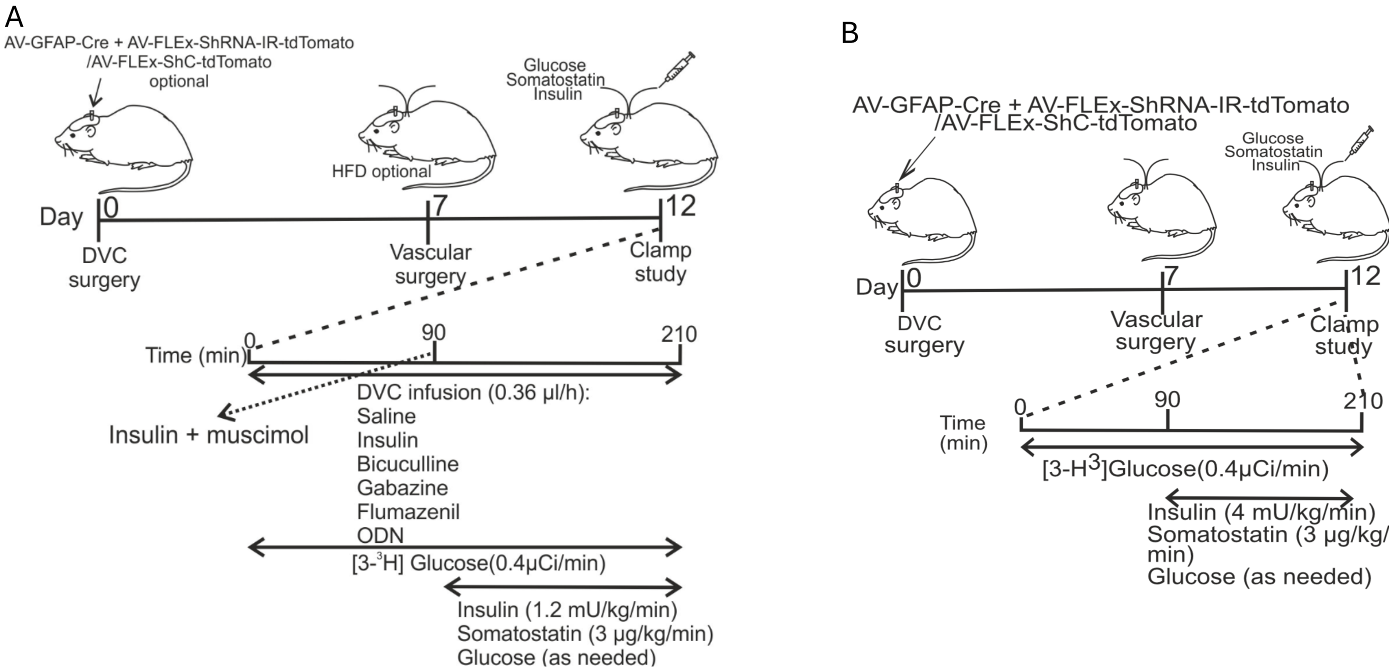


**Figure S4: A) Schematic of pancreatic-euglycemic clamp protocol. B) Schematic of Hyperinsulinemic clamp protocol.**

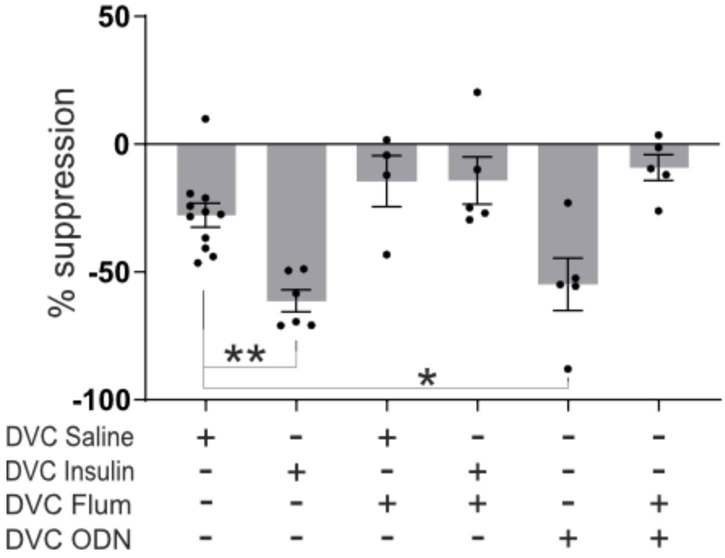


**Figure S5: Glucose kinetics during the clamps** with DVC flumazenil or ODN treatment in animals related to figure 4. Statistical analysis 1 -way ANOVA with Dunnett's post hoc comparing every condition with the saline. *p<0.05, ** p<0.01

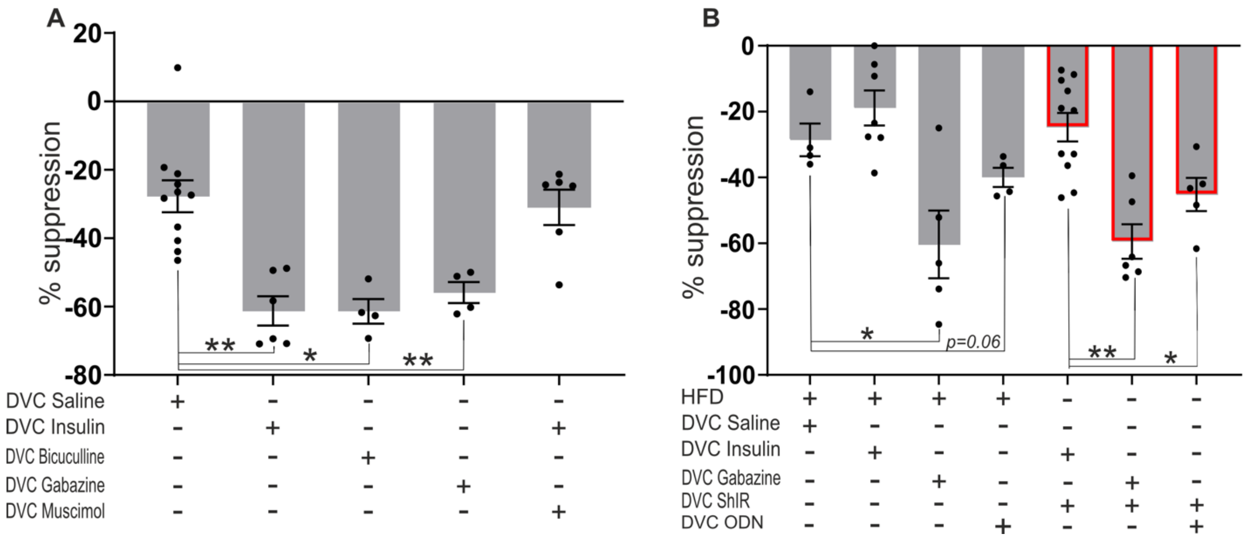


**Figure S6: Glucose kinetics during the clamps. (A)** Regular chow-fed animals treated with either DVC bicuculline, gabazine, muscimol treatment. Related to Figure 5Ai. **(B)** Glucose kinetics for either HFD fed or GFAP-ShIR-tdTomato+ animals treated with either gabazine, flumazenil, or ODN related to figure 5Bi. Statistical analysis 1 -way ANOVA with Dunnett's post hoc comparing every condition with the saline and A and with Saline or ShIR saline in B. *p<0.05, ** p<0.01

Table S1: Summary of basal and clamp insulin levels (ng/ml) during the pancreatic-clamp experiments

| DVC treatment | Basal insulin (ng/ml) (t-0-90) | Clamp insulin (ng/ml) (t150-210) |
|---|---|---|
| **Saline** | 1.4 ± 0.2 | 1.2 ± 0.1 |
| **Insulin** | 1.2 ± 0.1 | 1.1 ± 0.1 |
| **Bicuculline** | 1.2 ± 0.2 | 1.3 ± 0.04 |
| **GABAzine** | 1.0 ± 0.2 | 0.95 ± 0.07 |
| **Insulin + Muscimol** | 1.2 ± 0.22 | 1.1 ± 0.08 |
| **Saline (HFD fed)** | 2.2 ± 0.38 | 2.1 ± 0.38 |
| **Insulin (HFD fed) (p=0.4)** | 3.3 ± 0.77 | 2.6 ± 0.6 |
| **Gabazine (HFD fed)** | 3.1 ± 0.43 | 3.6 ± 0.27 |
| **ShC Saline (n=1)** | 2.0 | 2.0 |
| **ShC Insulin** | 1.5 ± 0.11 | 1.9 ± 0.52 |
| **ShIR Saline** | 1.2 ± 0.21 | 1.3 ± 0.1 |
| **ShIR Insulin** | 1.9 ± 0.44 | 1.6 ± 0.13 |
| **ShIR GABAzine** | 1.3 ± 0.29 | 0.92 ± 0.12 |
| **Flumazenil + saline** | 2 ± 0.19 | 2.1 ± 0.48 |
| **Flumazenil + insulin** | 2.3 ± 0.16 | 2.2 ± 0.5 |
| **ODN** | 2.6 ± 0.18 | 1.9 ± 0.2 |
| **Flumazenil + ODN** | 2.3 ± 0.26 | 2.1 ± 0.01 |
| **ShIR + ODN** | 2 ± 0.036 | 2.3 ± 0.3 |

Table S2: Summary of basal and clamp plasma glucose levels (mg/dl) during the pancreatic-clamp experiments.

| DVC treatment | Basal plasma glucose (mg/dl) (t0-90) | Clamp plasma glucose (mg/dl) (t90-210) |
|---|---|---|
| **Saline** | 134.6 ± 5.8 | 133.2 ± 5.6 |
| **Insulin** | 128.2 ± 10.1 | 124.3 ± 5.5 |
| **Bicuculline** | 125.0 ± 3.6 | 123.7 ± 5.8 |
| **Gabazine** | 141.5 ± 2.2 | 134.6 ± 2.9 |
| **Insulin + Muscimol** | 130.4 ± 4.9 | 131.6 ± 2.8 |
| **Saline (HFD fed)** | 137.2 ± 6.2 | 134.1 ± 8.4 |
| **Insulin (HFD fed)** | 141.2 ± 5.7 | 141.5 ± 9.7 |
| **Gabazine (HFD fed)** | 148.1 ± 5 | 146.4 ± 3.8 |
| **ShC Saline** | 138.3 ± 3.7 | 138.5 ± 6.7 |
| **ShC Insulin** | 149.6 ± 6.2 | 151.99 ± 10.5 |
| **ShIR Saline** | 149.6 ± 2.8 | 142.4 ± 4.6 |
| **ShIR Insulin** | 149.9 ± 4.6 | 147 ± 3.6 |
| **ShIR Gabazine** | 154.6 ± 5.4 | 148.6 ± 5.8 |
| **Flumazenil + saline** | 140.1 ± 1.9 | 144.3 ± 7.8 |
| **Flumazenil + insulin** | 143.1 ± 4.5 | 151.92 ± 6.9 |
| **ODN** | 138.5 ± 3.9 | 138.6 ± 3.9 |
| **Flumazenil + ODN** | 141.1 ± 3.1 | 149.2 ± 1.3 |
| **ShIR + ODN** | 140.6 ± 3.6 | 137.7 ± 5.2 |
| **Hyperinsulinemic clamp ShC** | 126.9 ± 4.21 | 128.4 ± 6.79 |
| **Hyperinsulinemic clamp ShIR** | 115.93 ± 6.95 | 121.77 ± 7.49 |
